# Arterial only anastomosis associated with modified Baudet technique in ear replantation: Case reports and literature review

**DOI:** 10.1097/MD.0000000000025357

**Published:** 2021-04-02

**Authors:** Mihaela Pertea, Petru Ciobanu, Vladimir Poroch, Natalia Velenciuc, Sorinel Lunca, Florin Anghelina, Dragos Octavian Palade

**Affiliations:** a University of Medicine and Pharmacy “Grigore T Popa”; b Clinic of Plastic Surgery and Reconstructive Microsurgery, “Sf. Spiridon” Emergency Hospital; c Clinic of Palliative Care; d Second Surgical Oncologic Clinic, Regional Institute of Oncology; e University of Medicine and Pharmacy of Craiova; f Clinic of Otorhinolaringology, “Sf. Spiridon” Emergency Hospital, Iasi Romania.

**Keywords:** amputation, ear, leeches, replantation

## Abstract

**Background:**

Total ear amputation is a relatively rare trauma with an absolute indication for surgical treatment. Numerous techniques for auricular reconstruction have been described. When local and general conditions allow microsurgical replantation, this must be the first choice. We propose the association of microsurgical techniques with some modification (modified Baudet technique) to obtain higher survival rate of the reimplanted stump.

**Methods:**

This study included cases of 3 male patients with total ear amputation, the injuries and their mechanism (workplace accident) being identical. Chief complaints were pain, bleeding, important emotional impact due by an unaesthetic appearance. The established diagnosis was traumatic complete ear amputation (grade IV auricular injury according to Weerda classification). Microsurgical replantation was performed only with arteriorraphy, and no vein anastomosis. Cartilage incisions and skin excisions were made to enlarge the cartilage-recipient site contact area. Medicinal leeches were used to treat venous congestion, to which systemic anticoagulant therapy was added.

**Results:**

The results showed the survival of the entire replanted segment in all cases, with good function and esthetical appearance. Patients were fully satisfied with the final outcome.

**Conclusion:**

Microsurgical replantation is the gold standard, for the surgical treatment of total ear amputation. We believe that cartilage incisions and the increased surface of contact between cartilage and recipient site has an adjuvant role in revascularization of the amputated stump (with only arterial anastomosis) and the use of hirudotherapy helps to relieve early venous congestion.

## 1 Introduction

The description of a “perfect” human face takes into account the relationships between its elements: eyes, ears, nose, and mouth.^[1]^ The human ear confirms to the shape of the Fibonacci spiral/helix sequence.^[2–4]^ The design channels the sound in a very directional and organized way. Thus, the ear is a structure with complex shape and function, playing an important role in facial esthetics.^[4]^ Its absence has a major emotional impact on the patient, and the reconstruction of such a structure is a real challenge for any surgeon. Total, isolated, post-traumatic ear amputation is not a common injury.^[5]^ Often, such an injury is associated with other types of traumas to the cephalic extremity, which will be brought to the fore. Total auricular amputation can result from animal bites, traffic or workplace accidents.^[6]^ Over time, the complexity of reconstruction has resulted in the description of many reconstructive techniques useful in cases of total or partial ear amputations. Nonvascularized reattachment of avulsed ears as a composite graft was reported as early as 1898.^[7,8]^ Unfortunately, the anatomical conditions, auricular vascularization, the size of the blood vessels at this level (0.3–0.7 mm) and the difficulty of identifying them and distinguishing arteries from veins, and last but not least the difficult access and the small size of the surgical field do not allow, in many of these cases, the performance of microanastomoses at the arterial, and especially venous level.^[9,10]^ That is why the described techniques aim at improving the vascularization in case of circulatory failure or insufficiency, and also at treating venous congestion due to the insufficiency of venorraphy or impossibility of performing it. The use of retroauricular and cervical flaps and of biochemical or medical leeches, respectively, proved beneficial in terms of the outcomes of ear replantation.^[11–13]^ At the end of 2017 at least 87 ear replantations were reported over 37 years, the first successful ear replantation being reported in the literature in 1980 by Pennington et al.^[14,15]^ We reported the cases of 3 male patients aged 45 to 58 years old with total ear amputation (grade IV auricular injury according to Weerda classification) following workplace accidents. In all 3 cases only arteriorraphy was performed, and in the immediate postoperative period medicinal leeches were used to treat venous congestion. To improve vascularization, besides arterial reconstruction, incisions were made into the cartilage (Baudet technique) associated with skin excisions for increasing the cartilage-recipient site contact area. In all cases, the outcome was good, with the survival of the entire replanted auricle.

## 2 Methods

We reported the cases of 3 male patients, all of them were the victims of similar workplace accidents resulted in total ear amputations. All three previously mentioned patients gave their consent to participate in this study and authorized the photographs for publishing. The approval of the Hospital Ethics Commission was obtained (for each of three cases), according to international regulations.

### 2.1 Case 1

A 45-year-old male patient, smoker for over 20 years, who was admitted in the Emergency Room 3 h after a wood-saw accident. The diagnosis at admission was work trauma with complete left ear amputation (grade IV ear injury according to Weerda classification) and left-hand metacarpal II and III closed fractures. The amputated segment was transported in good condition. Patient was informed about surgery, the technique used and the possible complications and failure of the surgery. The patient signed a written informed consent. Ear, Nose and Throat (ENT) examination did not identify associated injuries in the other ear segments. Emergency surgery was performed under general orothracheal anesthesia. For the microsurgical time, operating microscope, microsurgery instruments, and 10-0 nylon suture wires were used. For skin suture 5-0 nylon was used. Only one end-to-end arterial microanastomosis was performed. Venous anastomosis was not possible because the ends of the veins to be anastomosed could be identified concomitantly in the segment to be replanted and recipient area. Surgery began with the preparation of the amputated segment by debridement of the wound edges and identification of an arterial stump to perform anastomosis. The arterial stump at the level of the recipient site was identified and prepared, too. Next, we proceeded to the excision of an arc-shaped skin band in order to expose a larger cartilage surface. A similar skin excision (approximately 4 mm wide) was performed at the level of the remaining post-amputation defect. Thus, we obtained a wider contact surface of the cartilage with a larger well-vascularized bed and less tissue to be revascularized. Cartilage incisions were made at different depths and levels (as in the Baudet technique), some of them are transfixation incisions. End-to-end arterial microanastomosis was done with 10-0 nylon thread. A venous stump could not be identified in the replanting segment. Surgery was completed with 5-0 nylon skin suture with minimum stitches. Because of the poor blood flow to the ear, systemic anticoagulant treatment with weight-based heparin, dextran 40 and warm room was used. Venous congestion in the immediate postreplantation period, consequence of the absence of a draining vein, was treated with medicinal leeches. The application of medicinal leeches was initiated at 6 h postoperatively and was maintained for six days. The hirudotherapy was preceded by the psychological preparation of the patients, ruling out blood diseases, infections, hepatorenal diseases or malignancies, as well as a history of chronic treatments with anticoagulants or immunosuppressants. Between medicinal leech therapy (MLT) sessions heparin was administered locally. Antibiotic therapy was administered for the prophylaxis of infections due to the most often intense contamination of lesions but also for the prophylaxis of possible *Aeromonas hydrophila* infections due to the use of medicinal leeches. Ciprofloxacin 500 mg/day was used. Systemic anticoagulation treatment was also given in this case. The sutures were removed after 21 days. Hospitalization time was 10 days.

### 2.2 Case 2

**Figure 1:**
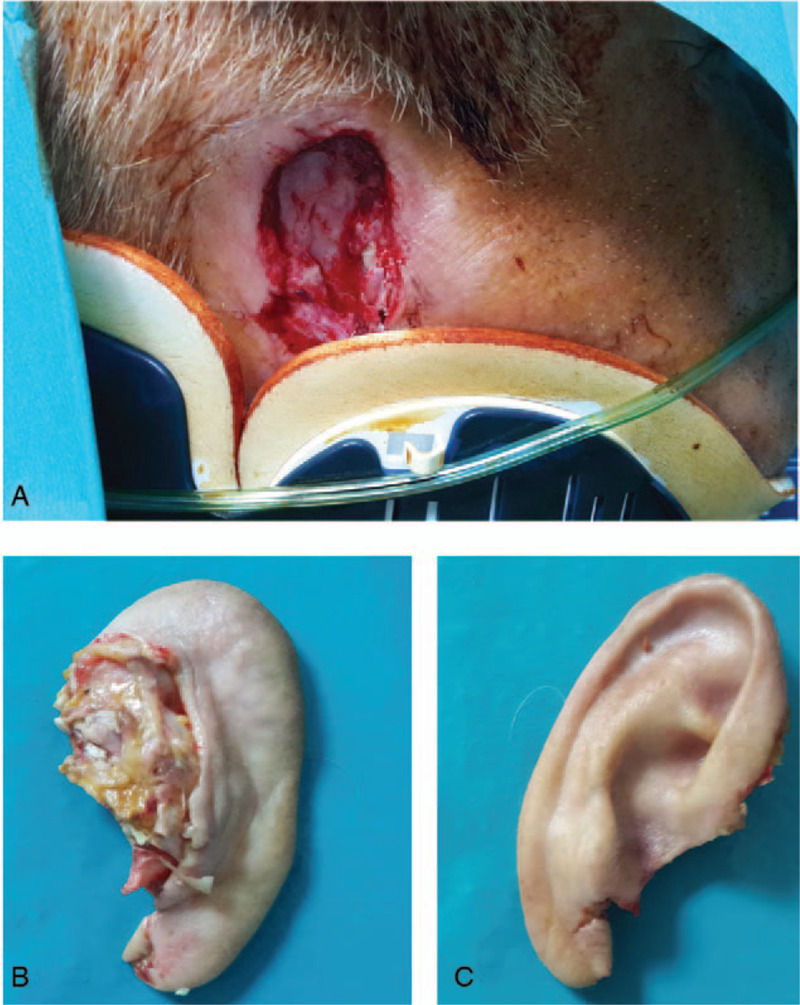
Total auricular amputation (A). Patient with total ear amputation and cervical spine contusion (with cervical collar) (B). Posterior aspect of the amputated stump (C). Anterior aspect of the amputated stump.

A 50-year-old male patient, non-smoker, who was admitted in the Emergency room at 2 h after a work accident (fall of concrete slab). The diagnosis at admission was work accident with complete right ear amputation, cervical spine cord contusion. The amputated segment was transported in a dry recipient. Patient was informed about surgery, the technique used and the possible complications and failure of the surgery. The patient signed a written informed consent. Ear, Nose and Throat (ENT) examination did not identify associated injuries. Neurosurgical examination allowed the emergency surgery under general anesthesia, recommending the permanent wearing of a cervical collar for 3 weeks. Surgery was performed under general orothracheal anesthesia. For the microsurgical procedure, operating microscope, microsurgery instruments, and 11-0 nylon suture wires were used. For skin suture 5-0 nylon was used. Only one end-to-end arterial microanastomosis was performed. Venous anastomosis was not possible because the stump of the vein to be anastomosed could be identified in the segment to be replanted. The wearing of the cervical collar throughout the microsurgical procedure brought additional discomfort to the surgical team, the patient position on the operating table and neck immobilization making the positioning of the operating microscope being extremely difficult (Fig. 1).

**Figure 2:**
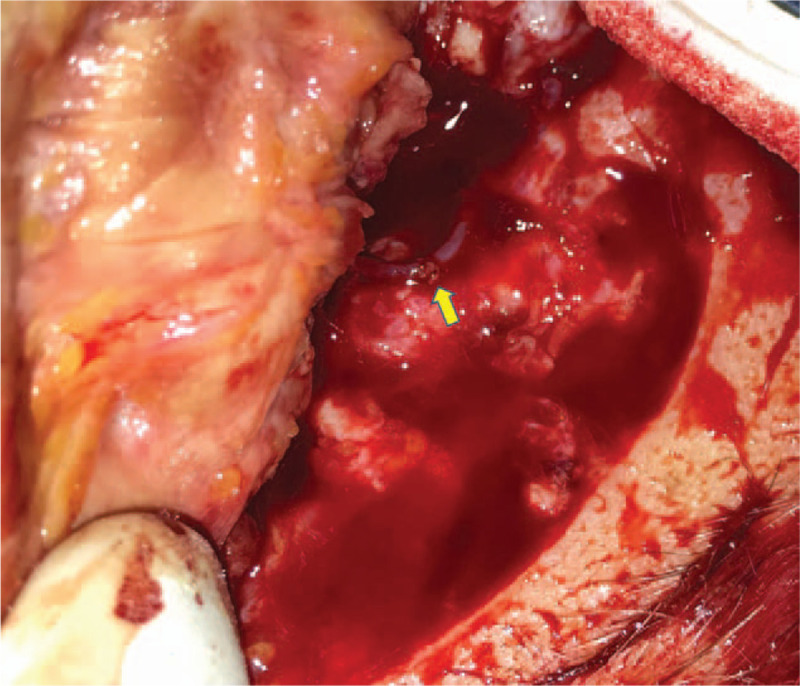
End-to-end arteriorraphy.

**Figure 3:**
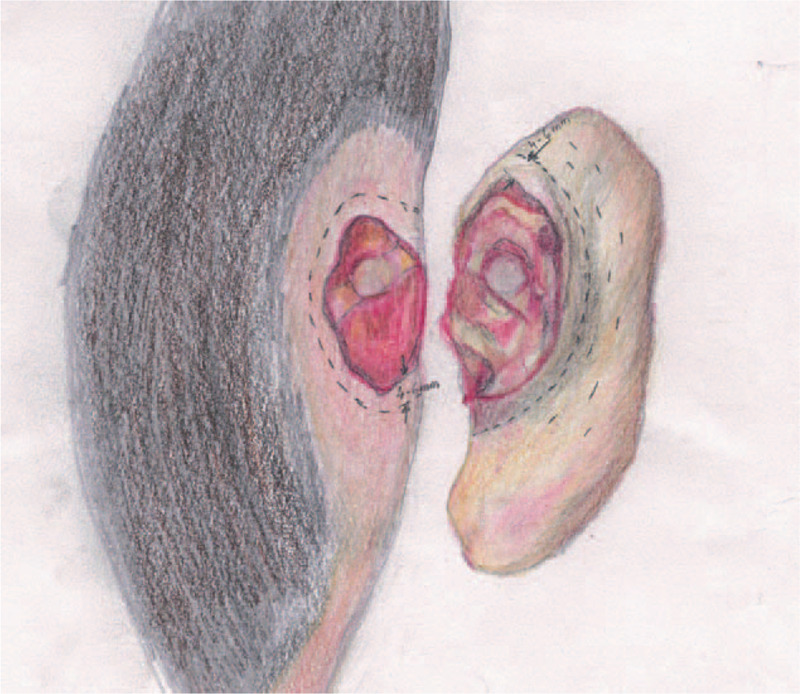
Surgical technique.

**Figure 4:**
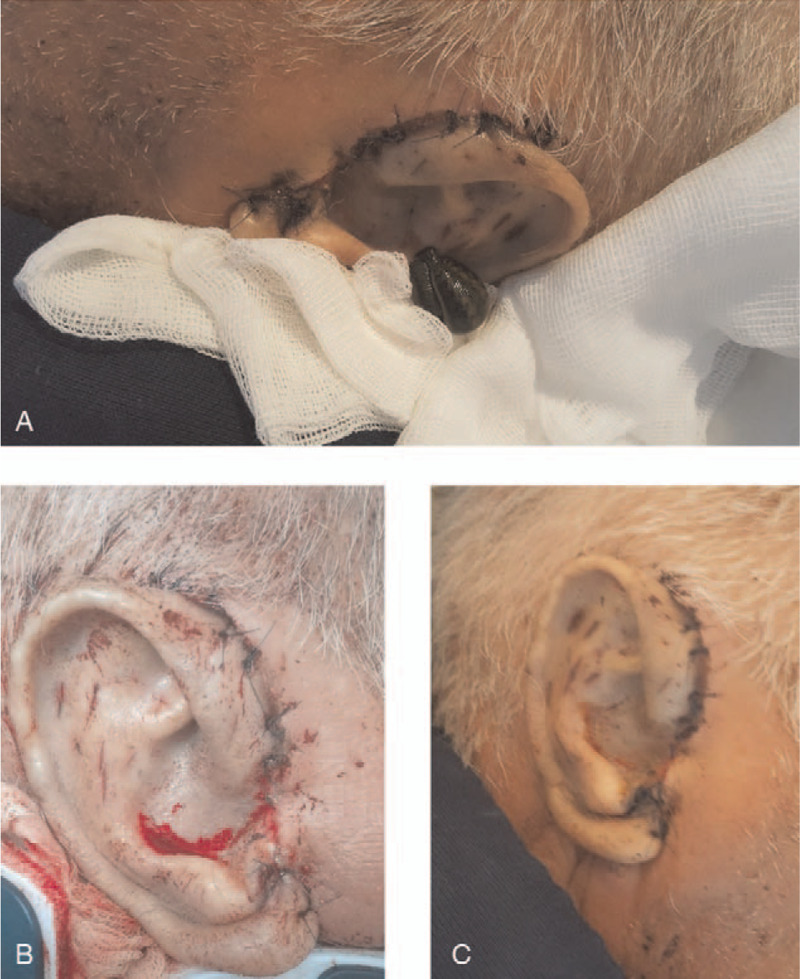
(A). Venous congestion of the replanted segment treated with medicinal leech therapy (hirudotherapy) (B). Appearance of the replanted ear after leeching on postoperative day 10 (C). Appearance of the replanted ear on postoperative day 21.

Surgery began, as in other cases, with the preparation of the amputated stump by debridement of the wound edges and identification and preparation of an arterial stumps to perform anastomosis. We proceeded to the excision of an arc-shaped skin band for exposing a larger cartilage surface. A similar skin excision was performed at the level of the recipient site. We obtained a larger contact surface of the cartilage with the recipient site. Cartilage incisions were made at different depths and levels (modified Baudet technique), some of them are transfixation incisions. End-to-end arterial microanastomosis was done with 10-0 nylon thread (Figs. 2 and 3). Surgery was completed with 5-0 nylon skin suture. The surgery lasted 6 h. We also used systemic anticoagulant treatment with weight-based heparin, dextran 40. At 6 h postoperatively, the first signs of venous congestion was reported. Medicinal leech therapy was started at 10 h postoperatively, after the psychological preparation of the patient and was continued for 5 days when there was no more venous congestion (Fig. 4). Hirudotherapy was supplemented by local administration of heparin. Systemic anticoagulation was also done. The sutures were removed after 14 days. Prolongation of operative time to 6 h was due to the technical difficulties related to patient and consequently of the position of the microscope, awkward posture of the surgeon, all related to the fact that patient had to wear the cervical collar. The patient remained hospitalized for a period of 12 days.

### 2.3 Case 3

A 58-year-old male patient, non-smoker, the victim of a work accident (falling from the same level and contact with a cutting object). He was admitted in the Emergency Room at 6 h after the accident. At the admission in the Plastic Surgery clinic the diagnosis was work accident with complete left ear amputation and sprained right ankle. No other ENT lesions was identified. The consultation of an orthopaedic surgeon was necessary. He indicated and performed the immobilization of the sprained ankle. The operative steps were similar as in the other two cases, using the same tools and the same sutures. We performed only one arterial anastomosis with no vein anastomosis. We also performed an arc-shaped skin band excision at both stumps (approximately 4 mm wide) to increase the contact area of the denuded cartilage with the recipient site. The surgery lasted 6 h. Medicinal leech therapy was started 12 h postoperatively and was continued for 7 days, when the signs of venous congestion disappeared. We used systemic anticoagulant treatment with weight-based heparin, dextran 40. Antibiotic therapy was administered, as in all cases, for the prophylaxis of infections due to the contamination of lesions but also for the prophylaxis of possible *Aeromonas hydrophila* infections. We also used ciprofloxacin 500 mg/day. The patient was discharged on the 11-th postoperatively day. The sutures were removed after 14 days.

In none of the study cases the great auricular nerve neurorrhaphy was performed.

Complete survival of the amputated segment was obtained in all cases, without areas of marginal necrosis or other complications that would require reintervention or subsequent secondary intervention for reconstruction. In none of the reported cases postoperative or leeching-related bleeding did not occur that required blood transfusions. No infections were recorded. During MLT, patients’ complete blood count (Hb, Ht) was repeated and secretions were collected from both the suture path and from where the leeches were placed. All 3 patients were fully satisfied with the results, as they regained their bodily integrity and esthetic appearance of the face. At the last follow-up visit 12 months after replantation all 3 reported cases showed esthetic appearance of the ear comparable with the normal contralateral ear, and even without primary neurorraphy, are covered a protective sensitivity of the replanted auricle (Table 1).

**Table 1:** Results.

	Sex	Age (y)	Surgical technique	Leeches used	Moment of leeches application after surgery	Anti coagulant therapy	Time of surgery (h)	Ischemia/necrosis	Blood transfusion	Infection	Viability of the replantated segment	Patient satisfaction
1	M	45	Only-artery anastomosis with cartilage incisions and skin excision	Yes	4 h	+	6	no	no	no	complete	Very satisfied
2	M	51		Yes	4,5 h	+	4	no	no	no	complete	Very satisfied
3	M	58		Yes	6 h	+	4,5	no	no	no	complete	Very satisfied

M = male, y = year, h = hours.

## 3 Discussions

The first microsurgical ear replantation was performed by Pennington in 1980, after Buncke announced in 1966 the first experimental microsurgical ear replantation.^[15,16]^ Over time, different classifications of ear trauma have been made.^[11,17]^ For clinical aspect Weerda proposed a classification into 4 grades.^[18]^ All cases included in the current report correspond to grade 4 in the Weerda classification. As to the non-microsurgical reconstruction of the ear, it has been done since 1898, when Brown, reattached the amputated segment as a composite graft.^[19]^ The psychological impact on the patient with such a trauma was very high and esthetics was the major concern. The importance of the ear as an essential element of facial esthetics, makes that in cases of total or partial ear amputation, the indication for replantation or reconstruction surgery to be an absolute one. That is why new reconstruction techniques have been reported over time, but microsurgical replantation remains the one with not only the best functional but also esthetic outcome.^[20–22]^ Although the techniques and used devices have evolved over time, microsurgical ear replantation is still a real challenge for any microsurgeon or plastic surgeon. In 1966 Buncke and Shultz performed the first ear replantation in a rabbit, and in 1980 Pennington reported the first successful replantation in a patient with total ear amputation.^[15,16]^ In 2005, Steffen published a 25-year review (1980–2004) of ear trauma. 37 of the review cases were total ear amputations in which microsurgical repair was performed. Of these, in 14 cases venorraphy was not performed. Total recovery rate when venorraphy was not performed was 5 out of 8 cases, while when venorraphy was performed it was 13 out of 18 cases.^[23]^ Because this type of lesion is quite rare, a systematic review conducted by Momeni and published in 2015 showed that in the interval 1980–2013 in 40 articles 60 cases of ear replantation after total amputation were reported. Of these, in 19 cases (31.7%) only arteriography was performed.^[24]^ Jung in 2011 also published a review of 52 replanted ears.^[25]^ At the end of 2017 Dvorak et al reported that at least 84 replantations had been described in the literature over a period of 37 years.^[14]^ In 2020 Gailey et al reported a review covering the interval 2017–2020 of 132 cases of ear amputations. Most of these patients were aged 20–40 years (48%), followed by those aged 40–60 years (23%), the same age range as in our study patients.^[6]^ It is assumed that the rarity of this type of trauma is due to the fact that it is often included in polytrauma, in which there may be injuries of much higher severity.^[14]^ In the current report, as in the other similar studies in the literature, this type of trauma is much more common among men. Good results have been also reported when replantation was performed 10 h after trauma.^[26]^ Microsurgical replantation in cases reported so far in the literature consisted of either restoring an artery and a vein, only an artery (as in our case reports), the use of a venous graft, arteriovenous shunt or arterialization of the venous system.^[27,28]^ A vein can be reconstructed by end to-end anastomosis, venous graft, or arteriovenous shunt. The success rate of a replantation when venorraphy is performed is 68%.^[14]^ Many authors choose to restore only one artery, and the treatment of postoperative venous congestion due to the impossibility of vein reconstruction or insufficiency or thrombosis of the reconstructed vein to be done by using biochemical or medicinal leeches.^[29–32]^ Venous congestion occurs in 75% of replantation cases. Venorraphy increases the operative time significantly but the chances of amputated stump survival are less. Classical replantation resulted in a replanted segment survival rate of 27%.^[33]^ The Baudet technique increased the chances of survival of the replanted auricle to 38%.^[34]^ Starting from the Baudet technique, using cartilage incisions, some of them transfixion incisions, we added to this procedure the increase in contact surface of the microsurgically transplanted cartilage with the well vascularized exposed (retroauricular) area (also obtained by excision of a skin island), which can increase the chances of cartilage revascularization, adding imbibition as in the case of the composite graft. By increasing the exposed cartilage area by the excision of a 4–6 mm-wide skin island, the amount of tissue to be revascularized has also decreased. As in the cases described in the literature so far, therapy for venous congestion consisted of the use of medicinal leeches concomitantly with biochemical leech at the level of transfixion incisions made at the level of the replanted cartilage.^[14]^ In all our three cases, the resulting bleeding did not require blood transfusion, although in about 50% of the reported cases blood transfusion was required due to significant bleeding.^[25]^ According to reports, the duration of surgery for ear replantation varies between 4 and 6 h as in the present study.^[24]^ As in the cases described in the literature, we administered systemic anticoagulant therapy without the combined use of aspirin.^[33]^ Prophylaxis by administering ciprofloxacin 500 mg/day prevented a possible leech-related *Aeromonas hydrophila* infection. As in the cases reported in the literature, the patients who underwent ear replantation surgery were fully satisfied with the outcome. Also, as in the reported studies in the absence of great auricular nerve neurorraphy one-year restoration of sensitivity was quite satisfactory.^[34]^

## 4 Conclusions

As with many other human body segments, the Fibonacci spiral/helix sequence, with its complex, difficult to reproduce structure, underpins the shape of the external human ear. The ear is essential in the esthetic appearance of a person, its absence implicitly have a psychological impact. So far, none of the multiple reconstructive surgical techniques for the amputated auricle has been able to fully reproduce the shape of the ear. Therefore, microsurgical replantation is the gold standard of surgical treatment in cases of total ear amputation. Hirudotherapy is the treatment for venous congestion with very good results. We believe that cartilage incisions and the increased surface of contact between denuded cartilage and receptor site has an adjuvant role in ear revascularization. In all cases of total or partial ear amputation, efforts must be made so that the patients are satisfied with the appearance.

## Author contributions

**Conceptualization:** Mihaela Pertea, Sorinel Lunca.

**Data curation:** Mihaela Pertea, Petru Ciobanu, Florin Anghelina, Dragos Octavian Palade.

**Formal analysis:** Petru Ciobanu, Vladimir Poroch, Natalia Velenciuc, Sorinel Lunca, Florin Anghelina.

**Investigation:** Vladimir Poroch, Natalia Velenciuc, Florin Anghelina.

**Methodology:** Mihaela Pertea, Petru Ciobanu, Vladimir Poroch, Natalia Velenciuc, Sorinel Lunca, Florin Anghelina.

**Software:** Petru Ciobanu, Vladimir Poroch, Florin Anghelina, Dragos Octavian Palade.

**Supervision:** Sorinel Lunca, Dragos Octavian Palade.

**Validation:** Mihaela Pertea, Vladimir Poroch, Natalia Velenciuc, Sorinel Lunca, Florin Anghelina, Dragos Octavian Palade.

**Visualization:** Petru Ciobanu, Vladimir Poroch, Dragos Octavian Palade.

**Writing – original draft:** Mihaela Pertea, Dragos Octavian Palade.

**Writing – review & editing:** Mihaela Pertea, Sorinel Lunca.
